# Functional Neuroimaging Investigations of Motor Networks in Tourette Syndrome

**DOI:** 10.3233/BEN-120281

**Published:** 2013

**Authors:** Aribert Rothenberger, Veit Roessner

**Affiliations:** ^1^Child and Adolescent PsychiatryUniversitymedicine GöttingenGöttingenGermany; ^2^Child and Adolescent PsychiatryTechnical University DresdenDresdenGermany

**Keywords:** Tic, Tourette, tic generation, motor network, neuroimaging, development

## Abstract

Motor and vocal tics are the core symptom of Tourette syndrome (TS). Tic generation seems to develop throughout the known motor pathways. This review focuses on functional neuroimaging in order to check this assumption. Also it elucidates the alterations and interactions of motor networks in TS depending on different contexts and circumstances like resting state, spontaneous tic movements, suppression of tics and premonitory urges, voluntary goal-oriented movements as well as electrophysiological neuronal stimulation. In general, the primary tic generating motor network uses the basic motor pathways differently, interacts with secondary sensorimotor networks and neuronal systems of cognitive behavioural control in a merely hierarchical manner, changing during neurodevelopment.

## 1. Introduction

The core symptoms of tic-disorders (including Tourette syndrome = TS) are motor and vocal tics as signs of a hypermotor disturbance. They can be described as fragments of normal motor or vocal behaviors. Hence, three questions may be raised: First, are such movements based on the usual and known motor circuits only and, second, if so, how might these motor networks be altered? Third, how might the basic pathophysiological background of tics be explained? Moreover, things have to be considered in a more complex way, since the altered functioning of these motor networks might be different depending on the state and context of the ongoing tic-activity (Fig. 1); i.e. does the patient find himself in a resting state without a tic, is he surprised by a spontaneous tic-movement, is he trying to suppress/ sculpture a premonitory urge or a tic and, finally, is he conducting a voluntary goal-directed movement or a tic-imitation? Different neuronal networks may be included in tic-generation and performance and interact for these kinds of behaviour. A basic motor-related Tic-Generating-Network (TGN) would be sufficient for the action of simple tics while for "tic + premonitory urge" and "tic + mental suppression" neurodynamics would go beyond the basic TGN. In order to investigate this neuronal networking with acceptable spatial and time resolution, functional neuroimaging seems to be a helpful tool and it is the focus of this narrative review to discuss its contribution related to the exploration of motor networks in TS.[1]

## 2. Resting state

While a patient with a severe tic-disorder usually shows a high frequency of prominent tics, only those persons with mild and less frequent tics may be in-

[1] Heterogeneity of samples (e.g. co-existing problems like ADHD, medication, gender, age, tic-profile, including/excluding premonitory urge, community vs. clinical sample) and methods (e.g. fMRI, MEG, EEG/ERP) and the influence of the related confounders on motor networks are not discussed in detail because of space limitation and lack of a relevant database for critical evaluation.

ISSN 0953-4180/13/$27.50 © 2013 – IOS Press and the authors. All rights reserved

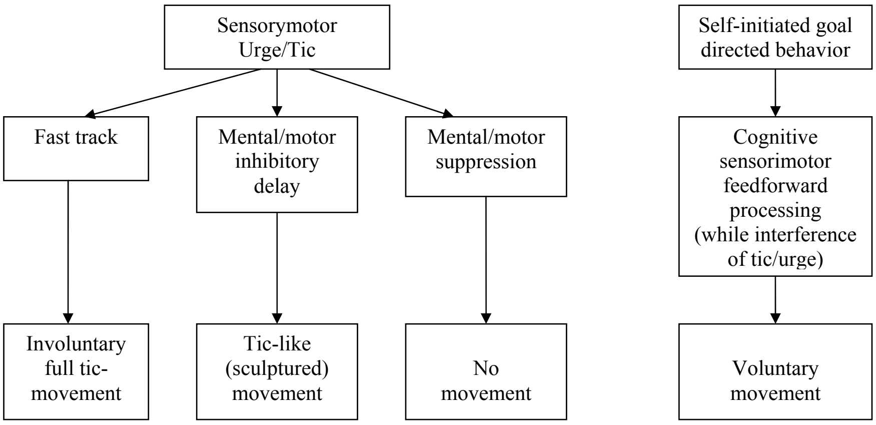

Fig. 1. Different kinds of behavioural functioning of motor networks in TS.

vestigated during a resting condition. Franzkowiak et al. [13] registered motor-cortical interaction in TS with magnetencephalography (MEG). They confessed that "a pure resting baseline level was not evident" (p. 4) and the "baseline period of 32 s rest following each task" by Werner et al. [54] using fMRI is also not a real resting state.

On the other hand, Serrien et al. [48], while testing functional activation patterns as revealed by EEG coherence, could clearly register data during an intermittend "resting" situation in TS, but they did not compare it with the one of healthy controls.

In their PET study to examine neuronal circuits of tic generation Lerner et al. [26] used stage 2 sleep as a baseline instead of the awake rest state. They wrote that the "...logic behind this approach is as follows: 1) Most movement disorders disappear during sleep. 2) The brain area generating the particular movement disorder is probably active most of the time during the waking state, independent of the presence or absence of the pathologic movement. However, the degree of activation may increase during generation of the particular pathologic movement. 3) Normally, regional brain metabolism does not differ much between wake and sleep stage 2." Unfortunately, for younger patients and/or fMRI sleep stage 2 seems to be not a feasible baseline condition/resting state. Further, tics occur also during sleep although less frequent and prominent [44].

Older studies (including experimental groups with a broader and less clear defined psychopathological profile) on 'baseline metabolism' in TS by FDG-PET showed an abnormal limbic-motor coupling compared with normal controls as well as overactivity of secondary motor areas [5,6,10,20].

In sum, there exists no clear information on the neurobiology of motor-networks in TS under resting conditions, especially, whether the functioning of these networks is altered already during these states and if yes how. Hopefully, the variability of individual behaviour during resting state (e.g. tic-suppression, muscle relaxation, concentration on something else, day dreaming) does reduce a possible bias of brain activation at a group level.

Probably, future EEG/ERP-studies with LORETA-analyses and coherence-analysis of the Default-Mode-Network or 'resting state' functional connectivity MRI (see [8,55]) might give more insight.

## 3. Spontaneous tic-movement vs. imitation

In their scholarly review on the neurobiology of TS Felling and Singer [12] included a short paragraph on functional neuroimaging and stated: "Event-related PET scanning has correlated tic occurrence with activity in a number of brain regions, including the prefrontal cortex, premotor and primary motor cortex, anterior cingulated cortex, putamen and caudate" [50]. However, the neurodynamics of tic generation still needs to be explored in more detail. Only two EEG studies [21,36] have registered the Bereitschaftspotential (BP; readiness potential) before spontaneous tics compared to the voluntary imitation of tic-movements. The absence of the BP before tics suggests that a different organisation of the motor network is active; i.e. in tics there is less involvement of mesial pre-motor areas, which might be more active with self-paced movements or control of non-acceptable movements.

Event-related functional MRI (fMRI) with synchronized video/audio recording techniques allows much better to explore the complete three-dimensional neuronal basis of single tics spontaneously exhibited by TS patients. Bohlhalter et al. [5] observed brain activities 2 s before and at the beginning of a simple tic. The authors identified a set of paralimbic brain areas (ACC and insular region) and parietal operculum activated before manifestation of single motor and vocal tics. Taken together with the PET-data of Stern et al. [50] and PET-results of Lerner et al. ([26], already cited as unpublished data in Bohlhalter et al. [5]) the latter suggested the constitution of a distinct neuronal network for tic generation (TGN) comprising insula, cerebellum, SMA and ACC plus parietal operculum. Unfortunately, Bohlhalter et al. [5] could not generate results on the imitation of tics because of methodological difficulties during registration. Further, it was not possible to differentiate brain activation between states of "pure" tics and tics accompanied by an urge which was clinically reported in about 50% of the TS patients. Therefore, we don't know which part of the suggested TGN goes beyond pure motor/vocal tic generation. Probably, two fMRI case-reports [14,22] might help to guide further group experiments. Kawohl et al. [22] found in their motor tic-condition also activation of the ACC and Gates et al. [14] during phonic tics activation in a broader TGN with parts of frontal, occipital, parietal and precentral cortex, nucleus caudatus, cingulated gyrus, cuneus and angular gyrus. In contrast, when a healthy person was mimicking the coprolalic phonic tics of the TS patient (unfortunately imitation by himself was not investigated) only right parietal cortex and left middle frontal gyrus were activated. Whether this indicated that mimicking tics (like in massed negative practice) does not touch the full TGN and thus limits this behavioural treatment option remains an open question.

A more valid approach are the group comparisons of Hampson et al. [17] and Wang et al. [53]. In both studies contrasts between spontaneous tics and intentional tic-like movements were investigated. Hampson et al. [17] followed the spatiotemporal patterns of coactivation between motor cortex and SMA before, during and after the movement in question. This coactivation was stronger for tics, while for both kinds of movements there was a nearly identical activation pattern of the other parts of motor-pathway. This highlights the central role of SMA for tic generation, probably the more the stronger a premonitory urge is present.

Wang et al. [53] also compared the activity of neuronal networks during spontaneous (TS patients) and simulated tics (healthy controls and TS patients). Their major findings were, first, a stronger neuronal activity in TS than in healthy controls throughout all portions of the motor pathway, second, stronger neuronal activity for spontaneous tics in a widespread cortico-subcortical network probably related to generation of tics plus premonitory urges and, third, a weaker activity of the cortico-striato-thalamo-cortical circuit (including caudate and ACC) reflecting reduced motor control for the movement of tic action although it is principally well preserved for general behaviour.

Both studies support the notion that tic generation may involve the usual motor pathway at least quantitatively different (e.g. enhances functional coupling between successive nodes). Further, tic generation also recruits other and interacts with other neuronal networks depending on tasks beyond motor/vocal tic generation.

## 4. Suppression of tics

Using fMRI Peterson et al. [37] showed that tic suppression is accompanied by increased activity in the right frontal cortex and right caudate while decreased activity could be detected in the globus pallidus, putamen and thalamus. These effects were inversely correlated with tic severity. Another study [2] found a positive correlation of tic severity with activation in substantia nigra and ventral tegmentum signalling that not only alterations of cortical and subcortical regions but also of brainstem loci may be involved in the dysfunctioning of motor networks in TS.

Usually, the voluntary suppression of tics is associated with sensorimotor phenomena (e.g. tension, pressure or tickle located in the very muscle group for a tic; or a diffuse tension somewhere in the brain or the body) leading to a strong urge for motor discharge which the patient tries to resist [1,46]. The latter can be used as a starting point for behavioural therapy with habit reversal training for TS [52]. Suffering from the triplet of sensorimotor pre-tic phenomena, mental effort to control the tics and the spontaneously initiated motor program of a pure tic-movement, obviously shows that the related neuronal network cannot be restricted to the basic motor network but needs a more complex neuronal interplay, especially if compulsive problems are involved. In adults with TS the latter are "correlated with functional abnormalities in associative and limbic networks, namely in orbito-frontal and prefrontal dorsolateral cortices" [55]. But also in children brain activation in cingulate gyrus, temporal gyrus and medial frontal gyrus may be correlated with obsessive-compulsive disorder score [9].

Jackson et al. [19] published a discussion paper "On the functional anatomy of the urge-for-action" and commented also on TS. They related the extended neuronal TGN as described by Bohlhalter et al. [5] to premonitory urges, because "electrical stimulation of the insular cortex or the parietal operculum can elicit unpleasant somatosensory or visceral sensations. By contrast, electrical stimulation of the medial frontal lobes produces motor outputs in the face and upper limbs comparable to tics . . . these two regions (remark by us: right insular cortex and mid-cingulate cortex bilaterally; see their Fig. 5) are linked functionally and can be thought of as the limbic sensory and motor areas". Probably, the key region for experiences of urges-of-action in TS is the insular cortex. This includes, that with brain development in tic children the awareness of tic-related sensorimotor phenomena (i.e. around the age of 10 years) other than the motor network are also involved in tic generation leading to a broader sensorimotor phenomenology. It remains to be considered which neuronal networks come additionally into play in order to supervise, monitor and control the tic-urge phenomenon. Berman et al. [3] suggested, on the basis of their experiments on blink suppression, that the right ventrolateral prefrontal cortex (VLPFC) with its involvement in inhibitory control plays a role in maintaining volitional suppression of blinking and other bodily urges, thoughts and behaviors. This is underlined by the co-activation of VLPFC with motor-associated cortices during movement inhibition.

## 5. Voluntary movement

For the earliest studies (in the 1980's) on spatial and temporal aspects of motor networks in TS only EEG-methodology was available. Studies were guided by the hypothesis that TS patients must prepare and execute a successful goal-directed movement in a different and more controlled way compared to healthy volunteers, because of interfering tics. Later Mink [31] suggested that voluntary movements might increase motor disinhibition in tic-disorders and thus induce facilitation of competing motor patterns more easily than in normals. However there is evidence even for a reduction of voluntary motor drive in TS [18].

At the 1980's registration of the BP allowed to investigate neuronal cortical activity before movement-onset (see also above under "tic-generation"). Rothenberger et al. [41–43] interpreted the increased shift of BP amplitude from central to frontal sites in tic children as an expression of their need to invest more mental effort into the control of a voluntary movement in order to achieve an adequate performance at a given task. Since the neuronal generator of the BP is related to SMA which is connected to the cingulated gyrus the authors suggested that this network must be involved in tic-control, an assumption which is confirmed and extended by todays studies on functional neuroimaging (e.g. [2,17,28]).

A recent publication [40] investigated right index finger tapping in 19 treatment-naive boys (age 12.5 years) with "pure" TS; the latter in order to avoid comorbid confounding factors and longterm compensatory brain effects. Compared to healthy controls, the children with "pure" TS activated the contralateral precentral gyrus to a lesser extent, suggesting "that TS patients might use a different neuronal recruitment strategy to execute simple motor tasks". In this study the statement was supported by the tendency of greater ipsilateral motor area deactivation and is in line with findings of less asymmetric BP data at lateral central sites (C3 and C4) for tic children [43] as well as with more diffuse fMRI activation of sensorimotor cortices and SMA [4]. Probably, this might be related to the thinner sensorimotor cortices and their altered degree of thinning in TS [11,49]. Finally, Roessner et al. [40] found also an increased left caudate nucleus activity. Deviations in the caudate/putamen are well known in TS. This focal excitatory abnormality may lead to thalamo-cortical dysrhythmia and neuronal disinhibition of the motor cortex as the last relais station followed by tic movements [12,25,30,31]. The additional recruitment of the caudate nucleus may be linked to more effort for motor control through fronto-striato-thalamo-cortical pathways.

Related to this Roessner et al. [40] registered a higher magnitude of activation in the right medial frontal cortex. The regulatory model of frontal cortex-basal ganglia interaction for motor control could be confirmed for tics over the years [29,41,43] and may be developed further in a more sophisticated way by functional neuroimaging within the near future. The same holds true for neurofeedback-training, which is based on this functional model, and showed preliminarily, that it can improve selfregulation of tics [46]. Finally, Roessner et al. [40] "observed decreased magnitude of activation in the right parietal, temporal and middle frontal cortices in TS". Hence, even during a simple finger tapping task in "pure" TS the involvement of neuronal networks goes beyond the basic motor network for voluntary movements. This indicates that tic-suppression and urges-for-action cannot be excluded fully in such experiments and seem to modify the alterations of motor networks in TS. Although the very strict methodology of this study allows some firm conclusions about the very early alterations of motor networks in the course of TS, it can finally not be decided "whether activation differences between both groups reflect functional compensating mechanisms or a primary disease process".

Werner et al. [54] investigated adults with TS and used also a sequential finger-tapping task. The alterations of motor network activation and functional connectivity in TS showed multilevel affection. Differential changes could be observed between groups (TS vs. normals) in cerebellum, brainstem, cingulum, temporal cortex, prefrontal cortex and parietal cortex, while functional clusters were depending on task performance and gradient of difficulty. This underlines, that because of heterogeneity between studies, it is difficult to find a simple and clear answer.

However, using a complex statistical approach Werner et al. [54] finally explained their findings with a lower-order motor network (brainstem, cerebellum, striatum) and a higher-order motor network (prefrontal areas, pre-SMA and parietal cortex) while the motor and sensorimotor cortex might be seen as an intermediate i.e. where Stop meets Go [51]. The latter cortices did not show up with group differences in the finger-tapping task of Werner et al. [54]. Nevertheless, during movement programming, motor preparation and execution there exists an important functional motor-cortical interaction between primary motor cortex and SMA. For example in preparation of a self-paced finger movement task Franzkowiak et al. [13] found this motor-cortical interaction increased in TS, reflected in neuromagnetic coherence at beta-frequency. After movement termination this coupling was no longer the case. This underlines that several alterations in motor networks of TS patients are strongly related to flexible, situational compensatory mechanisms which allow normal voluntary motor behaviour (see also [48] on EEG-coherence and motor inhibition).

Taken together, in both studies [40,54] most regions of the motor network showing deviations in structural neuroimaging were also altered functionally, although at the behavioural level no group differences for performance were seen and executive functions seem to be preserved in pure TS [16,39]. This doubts studies which assumed an impairment of fine motor skills in TS. At least, such findings may not directly result from tic-interference or an associated motor deficit but from factors such as medication and co-existing psychopathology. Moreover, even interhemispheric transfer within motor patterns seems to be well developed in TS, based on accelarated growth of callosal subregion 3 as a consequence of tic performance over the years [7].

In order to better disentangle and explain pure motor network alterations in TS from confounding circuits Worbe et al. [55] contrasted adult patients with only simple motor/vocal tics with two other groups having a more complex TS phenomenology including obsessive-compulsive traits. Their simple unilateral hand movements found only for the group with simple motor/vocal tics a reduction of activity in the primary motor cortex, but no group difference in the other clearly activated regions like SMA, ipsilateral cerebellum, posterior insula, putamen, thalamus, occipital cortex. On the one hand side, this clearly confirms that in TS the primary motor network is altered. On the other hand, it shows also that with reduced task load the other parts of the motor network as well as the merely compensatory/supervisory systems are functioning well in TS patients with only motor problems as could be seen in the results of a learning task in this study [55] and in tasks of inhibitory movement control [9,15,39,48].

It remains to be better clarified what happens if more difficult motor tasks have to be performed. For example, since basal ganglia mechanisms are underlying precision grip force control [38] it might be expected a behavioural deficit in TS patients, who may overscale their motor output and have difficulty coordinating grip and load forces [47]. In fact, their functional MRI showed underactivation in the secondary motor areas (premotor cortex, posterior parietal cortex, SMA) as well as in basal ganglia and thalamus compared to healthy controls. This is backed up with performance on inaccurate specification of the precision grip. While SM1 and cerebellum did not reveal any differences, the reported neuronal alterations are in line with the suggestion that a far reaching network is activated when not only voluntary movements but also interfering tics and premonitory urges (as both reported in these participants) are involved. Especially, a grip-load force control may activate compulsive behaviour/urges to touch objects forcefully as known in patients with complex TS.

Finally, it could be helpful to differentiate between networks active during early vs. late anticipation of a voluntary movement, since lower-order and higher-order systems are activated sequentially (i.e. first motor and premotor cortical areas and caudate nucleus are active while later ACC, frontal cortex and thalamus are involved) and might be differentially altered [27].

## 6. Electrophysiological brain stimulation

In order to assess neuronal excitability and thus functioning of the motor network in TS electrophysiological stimulation at different sites of the circuit is possible.

For example, transcranial magnetic stimulation (TMS) at the motor cortex (as a window for the whole motor circuit) showed that intracortical inhibition was deficient as seen by a shortened cortical silent period in TS patients [32,33,56]. The latter tends to normalize with age while tics are disappearing [34]. If the increased thinning of sensorimotor cortex in TS [11,49] is correlated to hyperexcitability of the motor system is not yet clear.

Another example is deep brain stimulation (DBS); e.g. at the thalamus or globus pallidus or anterior cingulum [35]. DBS may help to synchronize dysrhythmic thalamo-cortical oscillations driven by dysfunctional fast spiking neurons of the striatum [25].

Taken together, the different neuronal nodes of the motor network are interdependent and registered disturbances at one point of the network might reflect either a primary or a secondary problem or a compensation which went awry.

## 7. Perspectives

The reviewed studies have many methodological limitations which are only partly mentioned here but a detailed discussion would go beyond this report. Especially, the heterogeneity of samples in age and psychopathological profile presents a problem for generalisation of the findings. However, research in this area tries "to fail better".

So far, functional neuroimaging is on the way to better disentangle the different movement-related networks and their interplay involved and altered in TS. Because TS usually is composed not only of simple and complex motor/vocal tics but also of sensorimotor phenomena/urges with links to compulsions (e.g. touching, forceful grip) the basic (primary) motor network mostly interacts with secondary sensorimotor networks and neuronal systems of cognitive behavioural control and sensorimotor selfregulation. In addition, this complex interplay is changing during brain maturation. Age related abnormalities in frontostriatal and frontoparietal networks of comorbid TS patients exist [8,28,55] and lend support to the hypothesis that TS is a neurodevelopmental disorder [12]. But there exist good chances for a favourable outcome. Several compensatory and maturational mechanisms could already be shown (e.g. frontal lobe activation, interhemispheric callosal increase particularly of the motor subregion, improvement of cortical inhibition) and helps to better explain both etiopathophysiology and treatment principles.

Large scale developmental studies using functional neuroimaging combined with electrophysiology (like EEG/ERP, which may, in contrast to fMRI, differentiate between neuronal excitation and inhibition) and clinical treatment (with drugs or behavioural therapy) would be the next steps. Such an approach should find the right time-window during development for an intervention which improves best the actual problems as well as guarantees the optimal longterm outcome. Within this context it would also be interesting to know more about motor networks during sleep of patients with TS [23, 24,44]. This is all the more important as sleep without a tic-suppressing action has been discussed as the optimal baseline condition for fMRI studies on TS [26].

Finally, the feedback/feedforward mechanisms (including peripheral loops) concerning the awareness and control of tics should not be neglected [25,43].

## Box 1

The Tic-Generating-Network (TGN) and its Partner-Networks (PN) in functional neuroimaging of Tourette syndrome (T) – main findings depend on sensorimotor condition

- *Resting state*
  Methodological differences and differences between the few existing studies allow no firm conclusions on the neurobiology of motor-networks in TS under resting conditions
- *Spontaneous Tic-Movement vs. Imitation*
  Spontaneous tic generation may involve the usual motor pathway at least quantitatively different (e.g. less involvement of mesial pre-motor areas, enhanced functional coupling between SMA and motor cortex)
- *Suppression of tics and urges*
  Not only cortical and subcortical regions but also brainstem loci may be involved in the dysfunctioning of motor networks in TS. The extended TGN

may include as key regions the insular cortex as a limbic sensorimotor area and the ventrolateral prefrontal cortex as an area for inhibitory control

- *Voluntary movement*
  Patients with TS activate the basic motor network using a different (broader) neuronal recruitment strategy in order to execute motor tasks. At the same time they need to invest more mental effort (i.e. activating frontal lobes) to achieve an adequate performance. A regulatory model of frontal cortex-basal ganglia interaction for motor control could be confirmed for both voluntary movements and tics
- *Brain stimulation*
  Diagnostic and therapeutic use of electrical brain stimulation points to misguided brain oscillations in motor networks at cortical and subcortical neuronal areas.

*Comment*
All findings need to be refined according to brain development and tic-associated disorders. Several compensatory and maturational mechanisms could already be shown (e.g. improvement of cortical inhibition with age, callosal increase).

## References

[1] T. Banaschewski, W. Woerner and A. Rothenberger, Premonitory sensory phenomena and suppressibility of tics in Tourette Syndrome – developmental aspects in children, *Developmental Medicine and Child Neurology* **45** (2003), 700-703.

[2] C.L. Baym, B.A. Corbett, S.B. Wright and S.A. Bunge, Neural correlates of tic severity and cognitive control in children with Tourette syndrome, *Brain* **131** (2008), 165-179.

[3] B.D. Berman, S.G. Horovitz, B. Morel and M. Hallett, Neural correlates of blink suppression and the buildup of a natural bodily urge, *NeuroImage* **59** (2012), 1441-1450.

[4] B. Biswal, J. Ulmer, R. Krippendorf, H. Harsch, D. Daniels, J. Hyde et al., Abnormal cerebral activation associated with a motor task in Tourette syndrome, *American Journal of Neuroradiology* **19** (1998), 1509-1512.

[5] S. Bohlhalter, A. Goldfine, S. Matteson, G. Garraux, T. Hanakawa, K. Kansaku, R. Wurzman and M. Hallett, Neural correlates of tic generation in Tourette syndrome: an event-related functional MRI study, *Brain* **129** (2006), 2029-2037.

[6] A.R. Braun, B. Stoetter, C. Randolph, J.K. Hsiao, K. Vladar, J. Gernert, R.E. Carson, P. Herscovitch and T.N. Chase, The functional neuroanatomy of Tourette's syndrome: an FDG-PET study. I. Regional changes in cerebral glucose metabolism differentiating patients and controls, *Neuropsychopharmacology* **9** (1993), 277-291.

[7] J. Buse, J. August, N. Bock, D. Dörfel, A. Rothenberger and V. Roessner, Fine motor skills and interhemispheric transfer in treatment-naive male children with Tourette syndrome, *Developmental Medicine and Child Neurology* (2012), in press.

[8] J.A. Church, D.A. Fair, N.U.F. Dosenbach, A.L. Cohen, F.M. Miezin, S.E. Peterson and B.L. Schlaggar, Control networks in paediatric Tourette syndrome show immature and anomalous patterns of functional connectivity, *Brain* **132** (2009), 225-238.

[9] N.M.M.M. Debes, A. Hansen, L. Skov and H. Larsson, A functional magnetic resonance imaging study of a large clinical cohort of children with Tourette syndrome, *Journal of Child Neurology* **26** (2011), 560-569.

[10] D. Eidelberg, J. Moeller, A. Antonini, K. Kazumata, V. Dhawan, C. Budman et al., The metabolic anatomy of Tourette's syndrome, *Neurology* **48** (1997), 927-934.

[11] C. Fahim, U. Yoon, S. Das, O. Lyttelton, J. Chen, R. Arnaoutelis et al., Somatosensory-motor bodily representation cortical thinning in Tourette: effects of tic severity, age and gender, *Cortex* **46** (2010), 750-760.

[12] R.J. Felling and H.S. Singer, Neurobiology of Tourette syndrome: current status and need for further investigation, *Journal of Neuroscience* **31** (2011), 12387-12395.

[13] S. Franzkowiak, B. Pollok, K. Biermann-Ruben, M. Südmeyer, J. Paszek, T. Thomalla, M. Jonas, M. Orth, A. Münchau and A. Schnitzler, Motor-cortical interaction in Gilles de la Tourette syndrome, *PloSONE* **7** (2012), e27850.

[14] L. Gates, J.R. Clarke, A. Stokes, R. Somrjai, M. Jarmasz, R. Vandorpe and S.M. Dursun, Neuroanatomy of coprolalia in Tourette syndrome using functional magnetic resonance imaging, *Progress in Neuro-Psychopharmacology and Biological Psychiatry* **28** (2004), 397-400.

[15] A.M.Y. Goh, J.L. Bradshaw, J.A. Bradshaw and N. Georgiou-Karistianis, Inhibition of expected movements in Tourette's syndrome, *Journal of Clinical and Experimental Neuropsychology* **24** (2002), 1017-1031.

[16] E. Greimel, S. Wanderer, A. Rothenberger, B. Herpertz-Dahlmann, K. Konrad and V. Roessner, Attentional Performance in Children and Adolescents with Tic Disorder and Co-Occurring Attention-Deficit/Hyperactivity Disorder: New Insights from a 2x2 Factorial Design Study, *Journal of Abnormal Child Psychology*, (2012), in press.

[17] M. Hampson, F. Tokoglu, R.A. King, T. Constable and J.F. Leckman, Brain areas coactivating with motor cortex during chronic motor tics and intentional movments, *Biological Psychiatry* **65** (2009), 594-599.

[18] C.A. Heise, V. Wanschura, B. Albrecht, H. Uebel, V. Roessner, S. Himpel, W. Paulus, A. Rothenberger and F. Tergau, Voluntary motor drive (VMD) – possible reduction in Tourette syndrome, *Journal of Neural Transmission* **115** (2008), 857-861.

[19] S.R. Jackson, A. Parkinson, S.Y. Kim, M. Schuermann and S.B. Eickhoff, On the functional anatomy of the urge-for-action, *Cognitive Neurscience* **2** (2011), 227-257.

[20] K.J. Jeffries, C. Schooler, C. Schoenbach, P. Herscovitch, T.N. Chase and A.R. Braun, The functional neuroanatomy of Tourette's syndrome: an FDG PET study III: functional coupling of regional cerebral metabolic rates, *Neuropsychopharmacology* **27** (2002), 92-104.

[21] B. Karp, S. Porter, C. Toro and M. Hallett, Simple motor tics may be preceded by a premotor potential, *Journal of Neurology, Neurosurgery and Psychiatry* **61** (1996), 103-106.

[22] W. Kawohl, A, Brühl, G. Krowatschek, D. Ketteler and U. Herwig, Functional magnetic resonance imaging of tics and tic suppression in Gilles de la Tourette syndrome, *World Journal of Biological Psychiatry* **10** (2009), 567-570.

[23] R. Kirov, J. Kinkelbur, T. Banaschewski and A. Rothenberger, Sleep patterns in children with attention-deficit/hyperactivity disorder, tic disorder and comorbidity, *Journal of Child Psychology and Psychiatry* **48** (2007), 561-570.

[24] R. Kirov, T. Banaschewski, H. Uebel, J. Kinkelbur and A. Rothenberger, REM-sleep alterations in children with tic disorder and attention-deficit/hyperactivity disorder comorbidity: Impact of hypermotor symptoms, *European Child and Adolescent Psychiatry* **16**(suppl 1) (2007), i44-i50.

[25] J.F. Leckman, F.M. Vaccarino, P.S.A. Kalanithi and A. Rothenberger, Annotation: Tourette syndrome: a relentless drumbeat – driven by misguided brain oscillations, *Journal of Child Psychology and Psychiatry* **47** (2006), 537-550.

[26] A. Lerner, A. Bagic, E.A. Boudreau, T. Hanakawa, F. Pagan, Z. Mari, W. Bara-Jimenz, M. Aksu, G. Garraux, J.M. Simmons, S. Sato, D.L. Murphy and M. Hallett, Neuroimaging of neuronal circuits involved in tic generation in patients with Tourette syndrome, *Neurology* **68** (2007), 1979-1987.

[27] H. Luetcke, H. Gevensleben, B. Albrecht and J. Frahm, Brain networks involved in early versus late response anticipation and their relation to conflict processing, *Journal of Cognitive Neuroscience* **21** (2008), 2172-2184.

[28] R. Marsh, H. Zhu, R.T. Schultz, G. Quackenbush, J. Royal, P. Skudlarski and B.S. Peterson, A developmental fMRI study of self-regulatory control, *Human Brain Mapping* **27** (2007), 848-863.

[29] L. Mazzone, S. Yu, C. Blair, B. Gunter, Z. Wang, R. Marsh and B.S. Peterson, An fMRI study of frontostriatal circuits during the inhibition of eye blinking in persons with Tourette syndrome, *American Journal of Psychiatry* **167** (2010), 341-349.

[30] K. St. P. McNaught and J.W. Mink, Advances in understanding and treatment or Tourette syndrome, *Nature Reviews Neurology* **7** (2011), 667-676.

[31] J.W. Mink, The basal ganglia and involuntary movements: impaired inhibition of competing motor patterns, *Archives of Neurology* **60** (2003), 1365-1368.

[32] G.H. Moll, S. Wischer, H. Heinrich, F. Tergau, W. Paulus and A. Rothenberger, Deficient motor control in children with tic disorder: evidence from transcranial magnetic stimulation, *Neuroscience Letters* **272** (1999), 37-40.

[33] G.H. Moll, H. Heinrich, G.E. Trott, S. Wirth, N. Bock and A. Rothenberger, Children with comorbid attention-deficit hyperactivity disorder and tic disorder: Evidence for additive inhibitory deficits within the motor system, *Annals of Neurology* **49** (2001), 393-396.

[34] G.H. Moll, H. Heinrich, H. Gevensleben and A. Rothenberger, Tic distribution and inhibitory processes in the sensorimotor circuit during adolescence: A cross-sectional TMS study, *Neuroscience Letters* **403**(1–2) (2006), 96-99.

[35] K.R. Müller-Vahl, D.C. Cath, A.E. Cavanna, S. Dehning, M. Porta M.M. Robertson, V. Visser-Vandewalle and ESSTS Guidelines Group, European clinical guidelines for Tourette syndrome and other tic disorders. Part IV: Deep brain stimulation, *European Child and Adolescent Psychiatry* **20** (2011), 209-217.

[36] J.A. Obeso, J.C. Rothwell and C.D. Marsden, The neurophysiology of Tourette syndrome, in: *Gilles de la Tourette syndrome*, A.J. Friedhoff and T.N. Chase, eds, Raven, New York, 1982, pp. 105-114.

[37] B.S. Peterson, P. Skudlarski, A.W. Anderson, H. Zhang, J.C. Gatenby, C.M. Lacadie et al., A functional magnetic resonance imaging study of tic suppression in Tourette syndrome, *Archives of Genetic Psychiatry* **55** (1998), 326-333.

[38] J. Prodoehl, D. Corcos and D.E. Vaillancourt, Basal ganglia mechanisms underlying precision grip force control, *Neuroscience and Biobehavior Review* **33** (2009), 900-908.

[39] V. Roessner, B. Albrecht, P. Dechent, J. Baudewig and A. Rothenberger, Normal response inhibition in boys with Tourette syndrome, *Behavior and Brain Function* **4** (2008), 29.

[40] V. Roessner, M. Wittfoth, C. Schmidt-Samoa, A. Rothenberger, P. Dechent and J. Baudewig, Altered motor network recruitment during finger tapping in boys with Tourette syndrome, *Human Brain Mapping* **33** (2012), 666-675.

[41] A. Rothenberger, ed., *Event-related potentials in children*, Developments in Neurology, Vol 6, Elsevier, Amsterdam; 1982.

[42] A. Rothenberger, S. Kemmerling, G.K. Schenk, D. Zerbin and M. Voss, Movement-related potentials (MRPS) in children with hypermotoric behavior, in: W.C. McCallum, R. Zappoli and F. Denoth, eds, *Cerebral Psychophysiology: Studies in Event-Related Potentials, Electroencephalaography and Clinical Neurophysiology*, Suppl **38** (1986), 496-498.

[43] A. Rothenberger, The role of the frontal lobes in child psychiatric disorders, in: *Brain and Behavior in Child Psychiatry*, A. Rothenberger, ed., Springer, Berlin Heidelberg, 1990, pp. 34-58.

[44] A. Rothenberger, T. Kostanecka, J. Kinkelbur, S. Cohrs, W. Woerner and G. Hajak, Sleep and Tourette's syndrome, in: *Tourette Syndrome*, D.J. Cohen, J. Jankovic and C. Goetz, eds, Advances in Neurology, Vol. 85, Lippincott Williams and Wilkens, Philadelphia, 2001, pp. 245-259.

[45] A. Rothenberger, A systems approach to the brain basis of emotion also needs developmental and locationist views – the case of Tourette's syndrome, *Behavior and Brain Science* **35** (2012), 160.

[46] A. Rothenberger and H. Gevensleben, Selbstregulation von Tics – Optimierung durch Neurofeedback (Self regulation of tics – optimizing by neurofeedback), in: Strehl U, ed., Neurofeedback – ein Arbeitsbuch (Neurofeedback – a Handbook), (2012), in press.

[47] D.J. Serrien, A.C. Nirkko, T.J. Loher, K.-O. Lovblad, J.-M. Burgunder and M. Wiesendanger, Movement control of manipulative tasks in patients with Gilles de la Tourette syndrome, *Brain* **125** (2002), 290-300.

[48] D.J. Serrien, M. Orth, A.H. Evans, A.J. Lees and P. Brown, Motor inhibition in patients with Gilles de la Tourette syndrome: functional activation patterns as revealed by EEG coherence, *Brain* **128** (2005), 116-25.

[49] E.R. Sowell, E. Kan, J. Yoshii, P.M. Thompson, R. Bansal, D. Xu, A.W. Toga and B.S. Peterson, Thinning of sensorimotor cortices in children with Tourette syndrome, *Natural Neuroscience* **11** (2008), 637-639.

[50] E. Stern, D.A. Silbersweig, K.A. Chee; A. Holmes, M.M Robertson, M. Trimble; C.D. Frith, R.S. Franchowiak and J.R. Dolan, A functional neuroanatomy of tics in Tourette syndrome, *Archives of Genetic Psychiatry* **57** (2000), 741-748.

[51] C.M. Stinear, J.P. Coxon and W.D. Byblow, Primary motor cortex and movement prevention: Where Stop meets Go, *Neuroscience and Biobehavior Review* **33** (2009), 662-673.

[52] C. Verdellen, J. van de Griendt, A. Hartmann, T. Murphy and ESSTS Guidelines Group, European clinical guidelines for Tourette syndrome and other tic disorders. Part III: behavioural and psychosocial interventions, *European Child and Adolescent Psychiatry* **20** (2011), 197-207.

[53] Z. Wang, T. Maia, R. Marsh, T. Colibazzi, A. Gerber and B.S. Peterson, The neural circuits that generate tics in Tourette's syndrome, *American Journal of Psychiatry* **168** (2011), 1326-1337.

[54] C.J. Werner, T. Stöcker, T. Kellerman, J. Bath, M. Beldoch, F. Schneider, H.P. Wegener, J.N. Shah and I. Neuner, Altered motor network activation and functional connectiviy in adult Tourette syndrome, *Human Brain Mapping* **32** (2011), 2014-2026.

[55] Y. Worbe, C. Mahlherbe, A. Hartmann, M. Pélégrini-Issac, A. Messé, M. Vidailhet, S. Lehéricy and H. Benali, Functional immaturity of cortico-basal ganglia networks in Gilles de la Tourette syndrome, *Brain* **135** (2012), 1937-1946.

[56] U. Ziemann, W. Paulus and A. Rothenberger, Decreased motor inhibition in Tourette's disorder: evidence from transcranial magnetic stimulation, *American Journal of Psychiatry* **154** (1997), 1277-1284.
